# Evaluation of the surgical factor in postoperative pain control

**DOI:** 10.4103/1658-354X.71134

**Published:** 2010

**Authors:** Mohamed E. Shams, Hosam M. Atef

**Affiliations:** *Department of Surgery, Faculty of Medicine, Suez Canal University, Ismailia, Egypt*; 1 *Department of Anesthesia, Faculty of Medicine, Suez Canal University, Ismailia, Egypt*

**Keywords:** *Postoperative pain*, *impact of surgical factor*, *abdominal wall dissection*

## Abstract

**Background:**

Postoperative pain control has been studied extensively, including many perioperative pain control procedures. Unfortunately, the impact of the surgical technique was not objectively studied.

**Aim:**

The aim of this study is to evaluate if the type of surgical dissection needed for extensive abdominal wall dissection actually has an effect in the reduction of postoperative pain or not.

**Materials and Methods:**

Forty adult patients, 19 males and 21 females, were randomly divided into two groups with each group containing 20 patients having different varieties of anterior abdominal wall ventral hernia. Patients in group I had their hernias and abdominal wall flaps dissected by only sharp dissection using scalpel. Patients in group II had their hernias and abdominal wall flaps dissected using mainly blunt dissection assisted by sharp dissection where blunt dissection could not do the job. All the patients had general anesthesia. No preemptive analgesia was used. Nalbufen was used as the only postoperative pain killer and the total amount used of it was treated as the indicator for the intensity of postoperative pain.

**Results:**

The results of the present study showed that the total amount of Nalbufen used for the control of postoperative pain is significantly less in group I throughout the postoperative follow-up period.

**Conclusion:**

This study concludes that use of sharp dissection in cases of extensive abdominal wall dissection is statistically better than other methods of dissection in terms of postoperative pain control.

## INTRODUCTION

Pain is an unpleasant sensory and emotional experience associated with acute or potential tissue damage.[1] Unrelieved acute pain may lead to major harmful physiological and psychological effects, which may actually result in significant morbidity and even mortality.[2–4] In the contrary, effective acute postoperative pain relief directly results in decreasing morbidity and mortality, shortening of hospital stay postoperatively and improving patient satisfaction.[5–6]

Anesthesiologists had studied the role of different types of anesthesia and various types of anesthetics in the reduction of postoperative pain. Recently, pre-emptive analgesia has gained popularity and its role in the control of postoperative pain has been extensively studied.[7] Although it is well known that meticulous gentle surgical dissection can make minimal degree of tissue trauma, which directly affects the degree of postoperative pain,[8] unfortunately, the actual relationship between surgical dissection and degree of postoperative pain has not been objectively studied.

This work is a trial to study the relationship between different types of commonly used surgical dissection and the amount of postoperative analgesics needed to control postoperative pain – as an indication of the severity of postoperative pain – in patients with ventral hernias which actually need extensive abdominal wall dissection.

## MATERIALS AND METHODS

This was a prospective randomized controlled trial done in the Department of Surgery, Suez Canal University Hospital, Ismailia, Egypt. Forty adult patients, 19 males and 21 females, were randomly divided into two groups. Each group had 20 patients with different varieties of anterior abdominal wall ventral hernia. All the patients had their hernia repaired with anatomical facial repair, using monofilament nonabsorbable no. 1 suture material (Proline^®^), and reinforcement with on lay nonabsorbable mesh repair (Proline or Ethicon). Patients in group I had their hernia and abdominal wall flaps dissected using only sharp dissection using scalpel. Patients in group II had their hernia and abdominal wall flaps dissected using mainly blunt dissection assisted by sharp dissection where blunt dissection could not do the job. All the patients had strict hemostasis using coagulation diathermy. Complete medical history and physical examination were done for all patients including assessment of vital signs and airway assessment. Investigations included CBC, PT, PTT, random and fasting blood sugar, serum creatinine and ECG. Patients were ASA I or II. All patients had general anesthesia. standard monitors including ECG, HR, NIBP, TEMP and ETCO2. Thiopental (3-5 mg/kg)was used for induction. Atracurium. 5 mg/kg for tracheal intubation. then halothane for maintenance of general anesthesia, intraoperative analgesia by diclofenac sodium 1 mg/kg IM, no preemptive analgesia was used but all patients received 5 mg. Nalbuphine given after complete recovery and just before leaving the recovery room to the inpatient ward. No preemptive analgesia was used but all the patients received 5 mg Nalbufen which was given after complete recovery and just before leaving the recovery room to the inpatient ward. Twenty milligrams of Nalbufen was diluted in 10 ml 0.9 saline solution (2 mg/ml) for every patient and he/she was given 2 ml (4 mg) on patient request, with a maximum dose of 20 mg every 24 hours. If any patient still needed analgesia after this 20 mg of Nalbufen, he/she were given diclofenac sodium. The total amount of Nalbufen needed was calculated for every patient in 3, 6, 12, 24, 36, and 48 hours from the time of giving the postoperative dose of Nalbufen. The statistical analysis was done using the SAS software, including actual means (±SE), coefficient of variability (CV%), and least square analysis of variance test.

## RESULTS

The present study was conducted in the period between June 2003 and January 2005. Twenty patients were included in each study group; 11 males and 9 females in group I (sharp dissection) and 8 males and 12 females in group II (blunt dissection). The ages of the patients ranged between 35 and 55 years. The two groups were nearly matched, with all ventral hernias needing extensive abdominal wall flap dissection and all the patients used nonabsorbable Proline mesh, either 15×15 cm or 30×30 cm. The total dose of Nalbufen needed for control of postoperative pain on patient request was used as an indicator for the severity of pain in 3, 6, 12, 24, 36 and 48 hours in each group collectively.

The minimum, maximum and actual means (±SE) of total amount of Nalbufen used in every group are represented in Tables 1–3. The minimum, maximum and actual means of doses of Nalbufen needed for postoperative pain relief were significantly less in group I (sharp dissection) than in group II (blunt dissection) throughout the assumed period of postoperative follow-up, with minor inconsistent differences between males and females in both the groups. Most of the patients who had their hernia repaired with the use of sharp dissection (16 out of 20) did not need any analgesic during the first 3 hours after recovery.

**Table 1:** Means±SE of dosage of Nalbufen as an indicator for postoperative pain severity in patients having their ventral hernia repaired using sharp and blunt dissection in both sexes

Type of surgical dissection		Postoperative follow up time				
		3 hours	6 hours	12 hours	24 hours	48 hours
Sharp dissection	Male	1 (±0.65)	4.5 (±0.50)	6.5 (±0.73)	9.5 (±0.73)	16.5 (±0.91)
	Female	0.67 (±0.45)	4.67 (±0.45)	6.67 (±1.14)	10.33 (±1.25)	16.17 (±1.40)
	Overall	0.8 (±0.37)	4.6 (±0.33)	6.6 (±0.73)	10 (±0.79)	16.3 (±0.90)
Blunt dissection	Male	6.18 (±0.63)	9.45 (±0.81)	14.91 (±0.95)	19.27 (±0.49)	27.64 (±0.85)
	Female	5.33 (±0.67)	8.89 (±1.11)	14.22 (±0.97)	17.33 (±0.67)	26.22 (±1.90)
	Overall	5.8 (±0.46)	9.2 (±0.66)	14.6 (±0.67)	18.4 (±0.45)	27 (±0.96)

**Table 2:** Minimum of dosage of Nalbufen as an indicator for postoperative pain severity in patients having their ventral hernia repaired using sharp and blunt dissection in both sexes

Type of surgical dissection		Postoperative follow up time				
		3 hours	6 hours	12 hours	24 hours	48 hours
Sharp dissection	Male	0	4	4	8	12
	Female	0	4	4	4	8
	Overall	0	4	4	4	8
Blunt dissection	Male	4	4	8	16	24
	Female	4	4	12	16	20
	Overall	4	4	8	16	20

**Table 3:** Maximum dosage of Nalbufen (mg) as an indicator for postoperative pain severity in patients having their ventral hernia repaired using sharp and blunt dissection in both sexes

Type of surgical dissection		Postoperative follow up time				
		3 hours	6 hours	12 hours	24 hours	48 hours
Sharp dissection	Male	4	8	8	12	20
	Female	4	8	16	20	26
	Overall	4	8	16	20	26
Blunt dissection	Male	8	12	20	20	32
	Female	8	16	20	20	36
	Overall	8	16	20	20	36

Except for the first 3 hours of follow-up in group I, most values of CV% in both the groups throughout the postoperative follow-up period were around 25% [Table 4], and nearly all values of standard error were less than 15%. The reliability of actual means of doses of Nalbufen used for controlling postoperative pain in patients of both the study groups is shown in Table 3.

**Table 4:** Coefficient of variability of dosage of Nalbufen as an indicator for postoperative pain severity in patients having their ventral hernia repaired using sharp and blunt dissection in both sexes

Type of surgical dissection		Postoperative follow up time				
		3 hours	6 hours	12 hours	24 hours	48 hours
Sharp dissection	Male	185.16	31.43	31.85	21.79	15.54
	Female	233.55	33.36	59.08	41.95	30.05
	Overall	205.20	31.86	49.26	35.54	24.63
Blunt dissection	Male	33.79	28.52	21.09	8.40	10.14
	Female	37.50	37.50	20.3	11.54	21.72
	Overall	35.20	31.86	20.42	10.93	15.85

The analysis of variance test [Table 5] implies that the differences between the two study groups according to the type of surgical dissection, sharp and blunt, in the total amount of Nalbufen used for control of postoperative pain as an indication of the severity of postoperative pain, were highly significant all through the postoperative follow-up time, being significantly less in group I. At the same time, the effect of sex as well as the interaction between sex and type of dissection had consistently no statistically significant differences on the amount of Nalbufen used for control of postoperative pain in both study groups.

**Table 5:** Mean squares of least square analysis of variance of factors affecting the amount of Nalbufen needed for control of postoperative pain in patients having their ventral hernia repaired using sharp and blunt dissection in both sexes

Source of variability	Degree of freedom	Postoperative follow up time				
		3 hours	6 hours	12 hours	24 hours	48 hours
Type of dissection	1	236.36**	205.22**	621.098**	685.56**	1094.41**
Gender	1	3.403	0.388	0.659	2.981	7.441
Operative dissection * gender	1	0.647	1.307	1.775	18.735	2.847
Error	36	36	36	36	36	36
R-squared		0.668	0.513	0.636	0.712	0.642

* Statistically significant *P*≤0.005

** Highly statistically significant *P*≤0.0005

## DISCUSSION

It had been once said that the single most important factor in preventing infection is the gentleness of surgical technique, i.e., the minimization of tissue injury. However, sophisticated methods directed at reducing contamination, if the operative technique is poor, can lead to higher infection rate.[9] The same concept also applies perfectly for postoperative pain, i.e., whatever is the method of postoperative pain control, clumsy aggressive tissue manipulation increases the intensity and duration of postoperative pain. Although sharp dissection is the preferable and advisable method of dissection, blunt dissection is still used as a major type of dissecting the abdominal wall flaps in the repair of ventral hernia in some schools.

The present study was based on a clinical observation that patients who had their hernias done with sharp dissection did not ask for pain killers, as expected. Sex of the patients did not affect the amount of Nalbufen used for analgesia in both the groups. This actually ameliorates the sex effect on the results of this study as a source of bias. In the same time, the patients who had their flaps dissected by sharp dissection had used much lower amounts of Nalbufen for pain relief postoperatively than the patients who had their flaps dissected mainly by blunt dissection. The difference between the two groups was highly statistically significant. After exhaustive medical search throughout the internet, unfortunately, no papers were found reporting objectively the effect of surgical dissection on the level of postoperative pain in general surgery.

During sharp scalpel dissection, the nerves in between the abdominal wall flap and the muscles of the abdominal wall are sharply and cleanly cut, whereas while performing blunt dissection, most of the nerves are avulsed during surgery, especially the small nerve endings away from the main perforators, which should be identified and coagulated before cutting. Sharp nerve cut results in tidy axonal transaction. In the contrary, blunt dissection results in avulsion of the nerve axons; this may lead to more edema and bruises of the nerve sheaths, causing more pain sensation and more neuroma formation later on. Also, pain mediators are produced in lesser quantities with sharp dissection than with blunt dissection; this may reduce pain sensation even with operations involving large extent of dissection as in ventral hernias. Also, blunt dissection usually results in more tissue edema, more interstitial hematomas, and more inflammatory response; all these factors may lead to more pain sensation and increase the amount of pain killers needed for analgesia postoperatively.

This study concludes that sharp dissection is better than blunt dissection in the repair of ventral abdominal wall hernias, in terms of postoperative pain severity reflected by the amount of postoperative pain killers needed for postoperative analgesia.
